# The Long-Term Interfacial Evolution and Prediction of Carbon- and Glass-Fiber-Reinforced Epoxy Hybrid Rods under a Hygrothermal Environment

**DOI:** 10.3390/polym15102278

**Published:** 2023-05-12

**Authors:** Xiaodong Liu, Binwu Wang, Qingyong Su, Qingfu Zuo, Xiaopeng Song

**Affiliations:** School of Energy and Built Environment, Guilin University of Aerospace Technology, Guilin 541004, China; lxd@guat.edu.cn (X.L.);

**Keywords:** water absorption, performances evolution, degradation mechanism, life prediction

## Abstract

In order to promote the engineering applications of carbon- and glass-fiber-reinforced epoxy hybrid rods, it is necessary to fully understand its long-term hygrothermal durability. In the present study, the water absorption behaviors of a hybrid rod in a water immersion environment are studied experimentally, the degradation rules of the mechanical properties are obtained, and establishing a life prediction model is attempted. The water absorption of the hybrid rod confirms to the classical Fick’s diffusion model, and the water absorption concentration is determined by radial position, immersion temperature, and immersion time. In addition, the radial position of water molecules diffused into the rod is positively correlated with the diffusion concentration. The short-beam shear strength of the hybrid rod decreased significantly after 360 days of exposure; this is because water molecules interact with the polymer through hydrogen bonds to produce bound water during the immersion process, leading to resin matrix hydrolysis and plasticization, as well as interfacial debonding. In addition, the ingression of water molecules caused degradation in the viscoelastic behavior of the resin matrix in hybrid rods. The glass transition temperature of hybrid rods decreased by 17.4% after exposure at 80 °C for 360 days. The Arrhenius equation was used calculate the long-term life of short-beam shear strength in the actual service temperature based on the time–temperature equivalence theory. The stable strength retention for SBSS was found to be 69.38%, which is a useful durability design parameter for hybrid rods in civil engineering structures.

## 1. Introduction

The carbon-fiber (CF) and glass-fiber (GF) hybrid is an ideal method for increasing the mechanical properties of composites, and has gained a lot of attention in the last 30 years [1,2,3]. CF is a high-performance fiber, which has the advantages of a high strength and modulus, superior corrosion, and abrasion resistance [4]. Carbon-fiber reinforced polymer (CFRP) composites had widely used in the areas of aerospace, automotive manufacturing, sports equipment and civil engineer structures [5,6,7]. GF is another high-performance fiber with excellent electrical insulation properties, corrosion resistance, mechanical properties, thermal stability and low cost [8,9]. Glass-fiber reinforced polymer (GFRP) composites are applied in electrical insulation composites, construction, transportation tools, and wind power generation [10,11].

The main reasons for a hybrid of CF and GF are as follows: (1) Performance optimization: a hybrid of carbon fibers and glass fibers can achieve optimized properties; for example, the high strength and modulus of carbon fibers is complemented by the good electrical insulation properties and corrosion resistance of glass fibers [12]. This integrated optimization helps to increase the overall properties of hybrid composites in order to meet the needs of different applications. (2) Cost reduction: The high price of CFRP limits its large-scale application in engineering structures; in contrast, GFRP has a lower cost [13]. In the fiber hybrid, the cost of composites can be reduced while maintaining a high performance, making it more suitable for large-scale applications. (3) Resource utilization and environmental friendliness: CF- and GF-reinforced polymer (C/GFRP) composites can allow for performance advantages from both fibers [14], reduce the dependence on a single resource, and decrease carbon emissions [15]. (4) Innovative performance combinations: C/GFRP composites lead to new performance combinations and provide more possibilities for applications in different fields. For example, by changing parameters such as the fiber type, hybrid ratio, and production process, hybrid composites with new functions or properties can be developed to further broaden their application scope [16,17]. In conclusion, the hybrid of CF and GF combines the advantages of both fibers to achieve the goals of performance optimization, cost reduction, improved resource utilization, and environmental friendliness. In conclusion, the hybrid of CF and GF is a key method for increasing the performance of composites and is worthy of in-depth study.

In practical applications, the service environment will inevitably decrease the performance of composites [7,18]. Especially in the hydrothermal environment, the C/GFRP composite may undergo the ingression of water molecules, which may change the internal structure of the matrix and result in a decrease in mechanical performance, thermal stability, and so on [19]. Therefore, it is important to fully understand the water absorption and life degradation rules in hydrothermal environment before the developing and application of C/GFRP composites.

In recent years, a lot of researchers have studied the water absorption and life degradation rules of CFRP composites and GFRP composites. Many studies have shown that the water absorption of CFRP and GFRP composites in hydrothermal environments is related to the internal structure of the resin matrix [20,21], fiber–matrix interfacial properties [22,23], and environmental conditions [24,25]. Based on this, researchers have used various experimental methods and theoretical models to predict and evaluate the water absorption performance of FRP composites. In the case of CFRP composites, researchers have thoroughly investigated water absorption behaviors in hydrothermal environments, as well as the effect on mechanical performance and thermal stability. Similarly, the water absorption behavior and property changes of GFRP composites in water solution environments have received extensive attention. However, there are relatively few studies on the water absorption behavior of C/GFRP composites—systematic theoretical analyses and experimental investigations are still lacking. In addition, although there are many life evaluations for CFRP [26] or GFRP composites [27], there are still some limitations in the life evaluation of C/GFRP composites. This is mainly because the water absorption behavior and property changes of C/GFRP composites can be affected by a combination of factors, such as fiber type, hybrid ratio and interfacial properties. These limitations are mainly manifested in the difficulty of the existing models to accurately predict the performance and lifetime of hybrid composites under a complex service environment. Therefore, the life prediction methods for hybrid composites still need further research.

In this paper, the water absorption behaviors of C/GFRP rods in a water immersion environment were experimentally studied, the changes of their mechanical properties were analyzed, and establishing a life prediction model was attempted. The article firstly introduces the experimental methods, including information about the C/GFRP rods and water absorption experiments. Subsequently, short-beam shear experiments, scanning electron microscopy (SEM) analysis, and dynamic mechanical analysis (DMA) tests were used to investigate the changes in the thermal and mechanical performances of C/GFRP rods in a hydrothermal environment. Based on the experimental results, this paper explored the effect of water absorption behavior on the mechanical performances and attempted to develop a life prediction model for C/GFRP rods. This study aimed to obtain the durability design parameter for C/GFRP composites in practical applications.

## 2. Experimental Procedures

### 2.1. The Raw Materials Used in the Hybrid Rod

**Figure 1:**
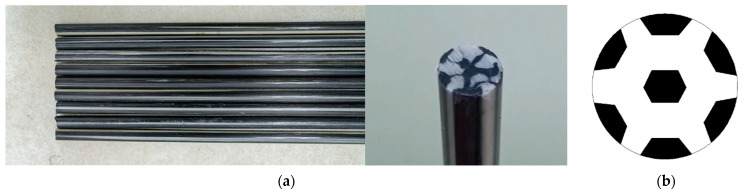
C/GFRP rod used in the experiment: (**a**) real production of rods and (**b**) fiber distribution.

Through fiber hybrid methods, C/GFRP rods were prepared using continuous pultrusion equipment (Harbin FRP Institute, Harbin, China). The diameter of the hybrid rods was 7 mm, and the rods produced could be up to several thousand meters long, the rods used in the experiment were obtained by cutting, as shown in Figure 1a. The detailed fiber distribution is shown in Figure 1b, the black areas represent carbon fiber and the white areas represent fiberglass. T700 carbon fiber (12K), Owens corning glass fiber (OC 2400tex), and anhydride-type epoxy resin matrix were used for the production of C/GFRP rods. The volume fraction of the fiber was about 70%, the volume fraction of the carbon fiber was 0.32. The detailed mechanical performances of fiber and matrix are list in Table 1.

### 2.2. Water Absorption and Desorption Behavior Testes

The water absorption test of the C/GFRP rod was conducted according to ASTM D5229M-14. When the length of the rod was larger than 30 times the diameter, it was considered that the water mainly experienced ingression along the rod radially, and the ingression along both ends of the rod could be ignored [28]. To be conservative, the C/GFRP rods were cut to 240 mm length in the present paper. Before testing, all of the samples were dried at 60 °C in an oven for one week to remove the original residual moisture. The specimens were exposed at 40 °C, 60 °C, and 80 °C with deionized water in other research work [19,29]. An electronic scale (Shanghai Jingke Industry Co., Ltd., Shanghai, China) with an accuracy of 0.1 mg was used to measure the mass change of water absorption periodically, and five specimens were used for each condition. After the measurement, the specimens were rapidly returned the water bath. Five specimens were tested for each condition, and the water absorption change of each specimen was calculated using the following equation:

(1) $$M_{t}=\frac{W_{t}-W_{0}}{W_{0}}\times 100$$

where *M_t_* is the percentage of water absorption, *W*_0_ is initial specimen weight, and *W_t_* is the exposed specimen weight.

The same specimen dimensions were used in the water desorption tests. The specimens were dried at 60 °C in an oven for 55 days and then at 120 °C in an oven for 35 days after exposure for 360 days. Five specimens were tested for each condition. The mass changes in water desorption after drying for each specimen were calculated using the following equation:

(2) $$M_{dt}=\frac{W_{dt}-W_{0}}{W_{0}}\times 100$$

where *M_dt_* is the percentage of water desorption and *W_dt_* is the dried specimen weight. It should be noticed that the surface of hybrid rod will inevitably suffer etching in the process of hygrothermal aging. If the mass of the rod is less than the original value when the rod is completely dried, *M_dt_* will be negative.

### 2.3. Short-Beam Shear Strength Test

**Figure 2:**
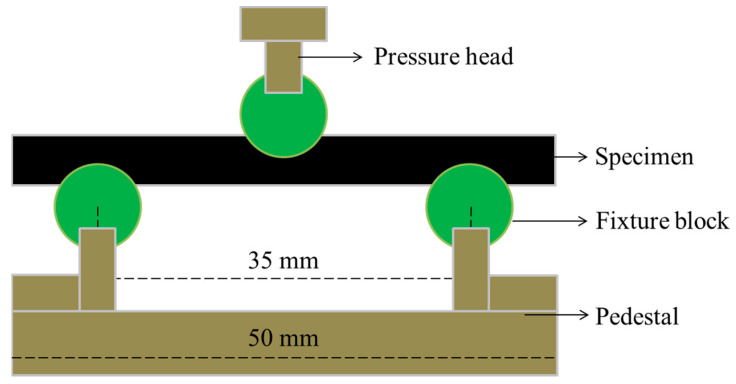
The short-beam shear test device diagram of C/GFRP rod.

The short-beam shear strength (SBSS) of the C/GFRP rods was calculated according to ASTM D4475. The hybrid rod was cut to 50 mm for the short-beam shear test samples and the span was 35 mm (five times the diameter of the rod). The universal testing machine (Shanghai LE5105 Precision Instrument Co., Ltd., Shanghai, China) was used to obtain the short-beam shear strength. The detailed short-beam shear test device diagram for C/GFRP rods is shown in Figure 2, where the displacement control is adopted. The displacement speed was set at 1.3 mm/min, and five samples were tested in each condition to obtain the average. The short-beam shear test for aging was carried out at intervals of 30, 90, 180, and 360 days on samples exposed to water.

### 2.4. Dynamic Thermal Mechanical Analysis (DMA)

The reference standard ASTM E1640−99 was used for the dynamic thermodynamic performance test of the exposed C/GFRP rods. The C/GFRP rod was cut into 35 mm × (5−10) mm × 2 mm using a precision cutting instrument (Secotom-50, Struers, Ltd., Ballerup, Denmark) Each condition tested two samples to obtain the average of T_g_. A dynamic thermodynamics tester was a dynamic mechanics analysis tester (DMA) produced by the TA Company in the United States, and the model is Q800. A single cantilever fixture type was used for the test, with a temperature rise rate of 5 °C/min, a temperature range of 25 to 250 °C, a collection frequency of 1 Hz, and an amplitude of 20 µm.

### 2.5. Microstructure Characterization

Scanning electron microscopy (SEM, VEGA3, Czech TESCAN, Czech) was used to investigate the microstructure of the C/GFRP rods before and after exposure. The dimensions of the specimens was 10 mm × 5 mm × 2 mm. Before the test, the sample was vacuumized and gold sprayed to improve its electrical conductivity. A frequency of 1000 Hz, 0.7 A of current, and 30 kV of voltage amplitude were selected in the test.

## 3. Results and Discussions

### 3.1. Water Absorption and Desorption

**Figure 3:**
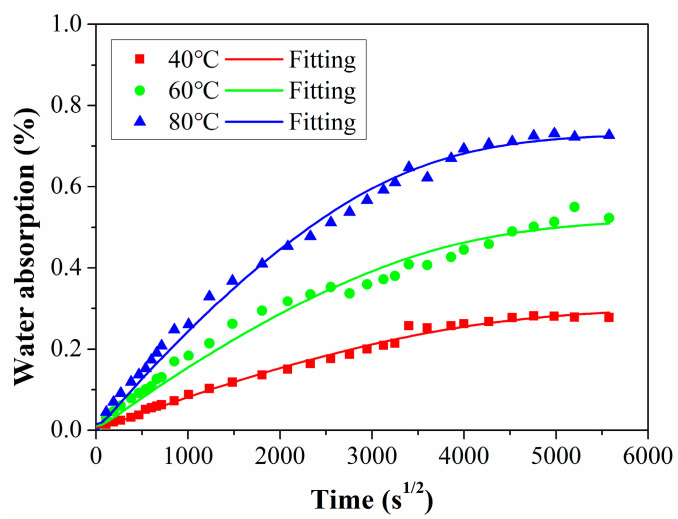
Water absorption curves immersed in distilled water at 40 °C, 60 °C, and 80 °C.

Figure 3 shows the water absorption of the C/GFRP rod increased gradually with the immersion time and temperature. After one year of exposure, the water absorption of the C/GFRP rods reached the maximum, namely 0.30% (40 °C), 0.52% (40 °C), and 0.72% (60 °C), respectively. The water absorption curve presented a nonlinear distribution overall and gradually tended to saturation. The results suggest that the water absorption characteristics of the C/GFRP rods were consistent with the classical Fick’s diffusion characteristics. In combination with the mathematical diffusion theory, Equation (3) was used to fit the water absorption test data of the C/GFRP rod, and the results are shown using the solid line in Figure 3.

(3) $$M_{t}=M_{\infty }\left[1-\sum_{n=1}^{\infty }\frac{4}{R^{2}\alpha_{n}^{2}}\exp(-D\alpha_{n}^{2}t)\right]$$

where *M*_∞_ is the percentage of the proposed equilibrium water absorption rate, *D* is the diffusion coefficient, *R* is the diameter of the C/GFRP rod, and *α_n_* is the nth root of the Bessel function of order zero.

**Figure 4:**
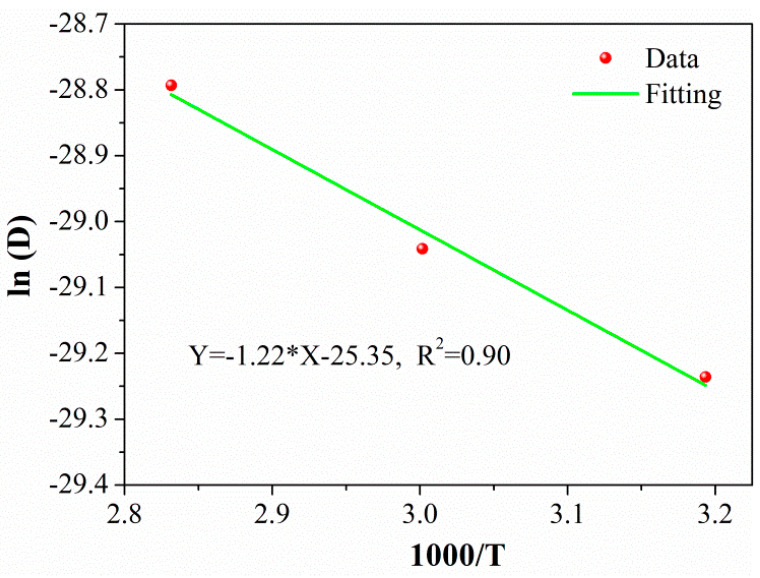
Linear relationship of ln (*D*) and 1000/*T*.

When the exposure temperature reached the glass transition temperature or reached the decomposition temperature of the resin, resin matrix cracking appeared inside the C/GFRP rod. Studies [30,31] have shown that when there was no cracking inside the C/GFRP rod, the total water absorption reached a certain value, independent of the exposure environment and time. For the water absorption performance of the C/GFRP rod, the maximum exposure temperature was 80 °C, and there was no external load. After exposure for one year, there was no obvious cracking inside the rod. Thus, it can be considered that the saturated water absorption rate of the C/GFRP rod in the above three exposure environments was a constant. According to the maximum water absorption rate and water absorption trend after one year of exposure shown in Figure 3, 0.72% was selected as the saturated water absorption rate of the C/GFRP rod. As shown in Table 2, the fitting degree of the water absorption curve of the rod body under three working conditions was higher than 0.98, indicating that the water absorption behavior of the C/GFRP rod body conformed to Fick’s law. Additionally, with the increase in immersion temperature from 40 °C to 80 °C, both the *M*_∞_ and *D* parameters increased. The main reason for this was that a higher temperature would increase the kinetic energy of water molecules, which could easily overcome the osmotic pressure of the C/GFRP rod. Based on the classical Arrhenius equation in Equation (4), the linear relationship of ln (D) and 1/T were established, as shown in Figure 4.

(4) $$D=D_{0}\exp(-\frac{E_{a}}{RT})$$

where *E_a_* is the activation energy, *R* is the gas constant (8.314 J/(mol*K)), *T* is the thermodynamic temperature, and *D*_0_ is the constant. It can be seen that the linear fit line fitted the experimental data well, demonstrating that the water absorption of the C/GFRP rod could meet the Arrhenius temperature acceleration requirement.

**Figure 5:**
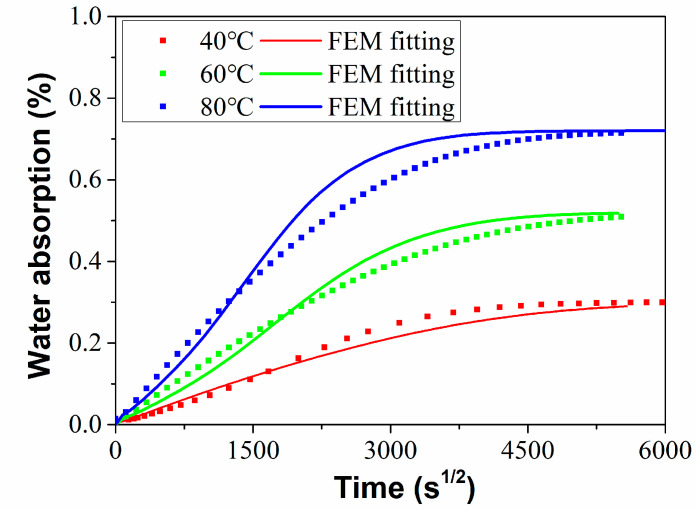
Comparison of the experimental and simulation results for C/GFRP rods.

To further analyze the diffusion process of the water molecules in C/GFRP rods, the finite element model (FEM) was established using ABAQUS software. The process of water absorption in the ABAQUS finite element software was simulated using the mass diffusion module. The material parameters in Table 1 were input into the model to obtain the water absorption curves, which were compared with the experimental test results, as shown in Figure 5. It can be found that the simulation results fitted well with the experimental results, which proved the correctness of the finite element model for water absorption.

To analyze the radial distribution of water molecules in C/GFRP rods, the diffusion mathematical theory was used to quantitatively characterize the influence of exposure temperature on the distribution of water molecules. For the radial diffusion of a long cylinder, the diffusion equation [32] is as follows:

(5) $$\frac{\partial C(r,t)}{\partial t}=\frac{1}{r}\frac{\partial }{\partial t}(rD\frac{\partial C(r,t)}{\partial r})$$

where *C*(*r,t*) is the concentration distribution related to the radial position and exposure time, *r* is the C/GFRP rod radius (mm), *t* is the water absorption time of the C/GFRP rod (s), and *D* is the average diffusion coefficient of the C/GFRP rod.

**Figure 6:**
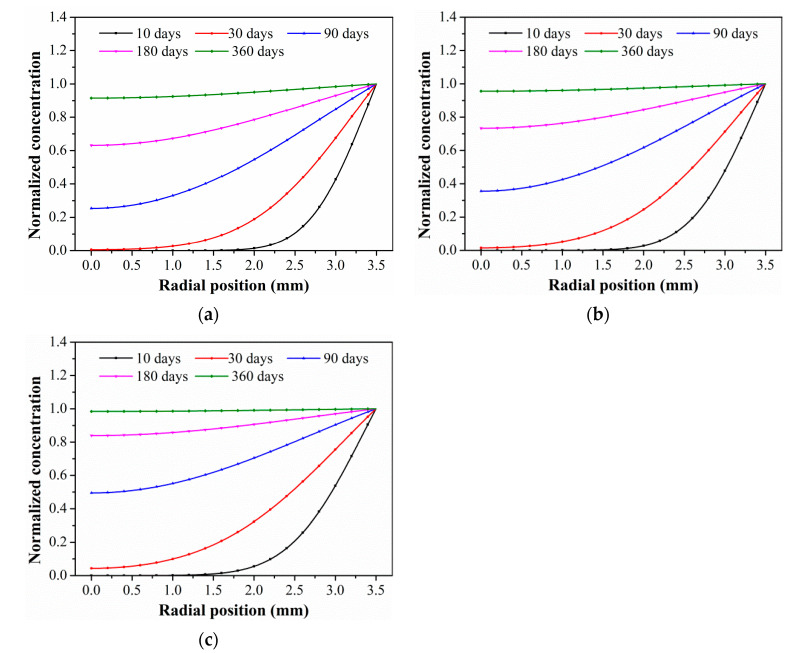
The variations in water absorption concentration for a C/GFRP rod with a radial position at a specific exposure time: (**a**) 40 °C, (**b**) 60 °C, (**c**) 80 °C.

Based on Equation (5), the water absorption concentration distribution of the C/GFRP rod was obtained as a function of the radial position, immersion time, and temperature. Figure 6 shows the radial distribution curve of the absorption concentration of the C/GFRP rod immersed at 40 °C, 60 °C, and 80 °C. The absorption concentration of the C/GFRP rod increased greatly with the exposure temperature, especially in the early stage of exposure. The main reason was that the diffusion rate of water molecules along the radial direction of the C/GFRP rod increased up at a higher temperature. Additionally, the water absorption concentration increased with the increase in radial position. Furthermore, the saturated water absorption of the C/GFRP rod was higher than 360 days at 40 °C and 60 °C, and about 360 days at 80 °C. Therefore, for saturated water absorption at the same radial position, the exposure temperature was a key factor for the saturated water absorption time.

The radial water absorption concentration distribution of the corresponding time nodes in each of the above exposure environments was obtained through the finite element model, as shown in Table 3. It can be seen from Table 3 that the diffusion behavior of the water molecules in the rod is consistent with results of Figure 5. The cloud chart of water distribution in the early stage changed obviously, because the diffusion speed of water molecules in the rod was relatively fast in the early stage. In addition, the distribution range of moisture in the cross-section of the rod widened at the same time as the immersion temperature. In other words, temperature accelerated the diffusion process. Therefore, the degree of diffusion for water molecules increased along with time and temperature.

**Figure 7:**
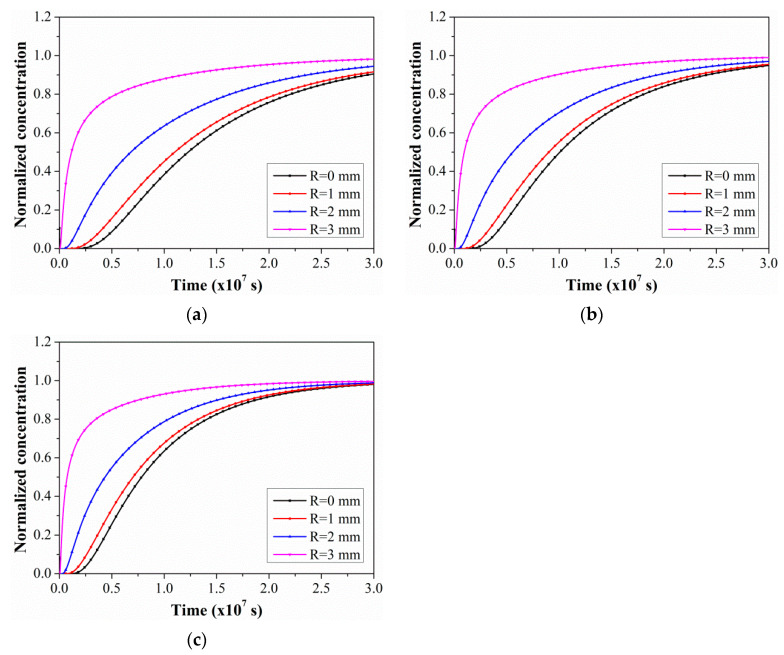
The variations in the water absorption concentration of C/GFRP rods with exposure time at specific radial positions: (**a**) 40 °C, (**b**) 60 °C, and (**c**) 80 °C.

To further analyze the evolution law of the C/GFRP rod water absorption concentration along the radial position under different exposure times and temperatures, Figure 7 shows the variations in water absorption concentration for the rod with exposure time at specific radial positions. According to Equation (5), four locations in the core layer center (r = 0.0 mm), inner core layer (r = 1.0 mm), central layer (r = 2.0 mm), and cortex (r = 3.0 mm) were selected in the rod to analyze the changes in water absorption concentration with exposure time. As shown, the water absorption concentration increased significantly with the radial position at the same exposure temperature and time, and the elevated temperature reduced the time taken to reach the saturation concentrations.

To quantitatively analyze the water absorption rate of the C/GFRP rod, the initial water absorption time of the rod was selected when the water absorption concentration was 0.01, and the saturation water absorption time of the rod was selected when the water absorption concentration was 0.99, as shown in Table 4. When the water absorption concentration at the central position of the C/GFRP rod (r = 0.0 mm) was 1.0, the time taken was called the saturated water absorption time. It was found that the initial and saturated water absorption time of the hybrid rod decreased gradually with the exposure temperature and radial position. For example, for the radial position of 3 mm, the saturated water absorption times of the C/GFRP rod bodies were 418.13, 342.78, and 267.08 days for 40 °C, 60 °C, and 80 °C, respectively. For comparison, with a decrease in radial position from 3 mm to 0 mm, the saturated water absorption time of the C/GFRP rod increased by 48.83%, 48.82%, and 48.88%, which indicated that the rate of water molecules diffusing into the hybrid rod was uniform, and the exposure temperature only increased the acceleration of diffusion for the water molecules. In conclusion, the exposure temperature was the main factor affecting the water absorption and diffusion behavior in the C/GFRP rod.

**Figure 8:**
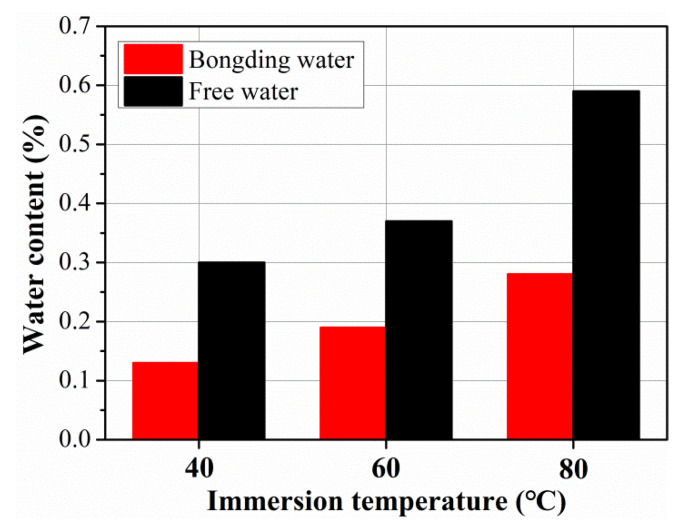
The content changes for the free and bonding water after 360 days of exposure at different temperatures.

The free and bonding water contents for the C/GFRP rod at three exposure temperatures are shown in Figure 8, and the specifications of the free and bonding water in the rod after 360 days of exposure was referenced from the research of [33]. Based on the total saturated water (Figure 3), it can be seen that the drying water content was greater than the water absorption content, indicating that the C/GFRP rod suffered mass loss during the exposure process because the resin matrix had been etched and the fibers/resin interface had de-bonded. Additionally, it is worth noting that the content of free water in the C/GFRP rod was greater than that of the bonding water. For example, the detailed contents of the free and bonding water were 0.30 and 0.13 at 40 °C, 0.37 and 0.19 at 60 °C, and 0.59 and 0.28 at 80 °C. Furthermore, the content of the free and bonding water increased with the exposure temperature, which, because of the elevated exposure temperature of 80 °C, increased the diffusion rate of the water molecules.

**Figure 9:**
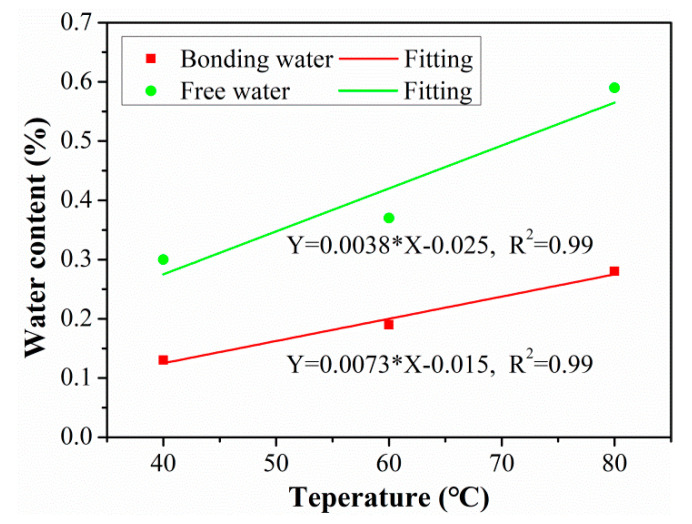
Relationships between the free water content, bonding water content, and exposure temperature.

In order to clarify the quantitative effect of temperature on water content, the relationship between free water content, bonding water content, and exposure temperature was established, as shown in Figure 9. It can be concluded that the content of free water diffusion in the C/GFRP rod was about twice as much as that of the bonding water for the three exposure temperatures. This suggests that most of the water molecules in the C/GFRP rod were in a free state, which played a role in lubricating the polymer chain of the resin matrix. In contrast, bonding water was the key factor causing the hygrothermal aging of the C/GFRP rods. This was because the water molecules existing in the state of bonding water would break the three-dimensional cross-linking between the resin chains in the form of a hydrogen bond with the resin matrix, resulting in the decomposition of the resin matrix chain and debonding of the fiber–resin interface. In summary, the content of free and bonding water increased linearly with the exposure temperature, with the slope of free water being greater than that of the bonding water, because the procedure for bonding water was harder to perform than for free water.

### 3.2. The Degradation of Short-Beam Shear Properties

**Figure 10:**
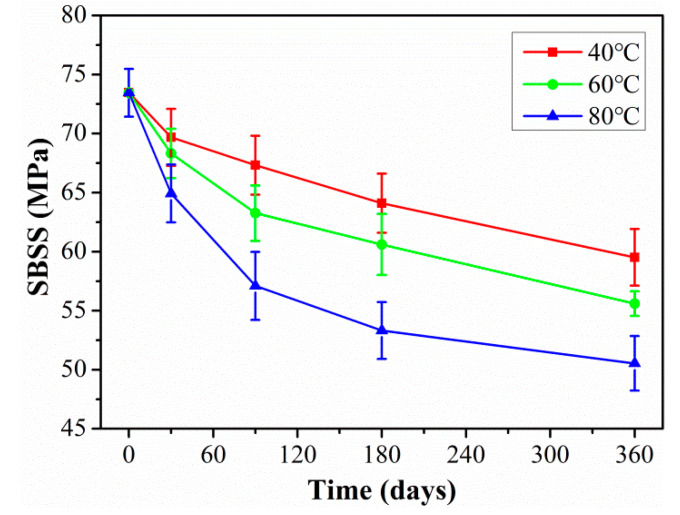
Variations of SBSS exposed for 360 days at 40 °C, 60 °C, and 80 °C.

Figure 10 shows the SBSS of C/GFRP rods exposed to distilled water for 360 days at 40 °C, 60 °C, and 80 °C. It was found that the SBSS of C/GFRP decreased with exposure time, especially for 80 °C. For example, compared with the SBSS (73.1 MPa) of the control C/GFRP, the SBSS after 360 days of exposure decreased by 14.7% for 40 °C, 21.3% for 60 °C, and 29.4% for 80 °C. The decrease in SBSS can be attributed to the fact that water molecules bonded with the resin matrix through hydrogen bonding to form two kinds of bonded water, which resulted in the plasticization and hydrolysis of the resin matrix and fiber–resin interface debonding, causing a decrease in fiber–resin interface shear strength. Additionally, it was noted that the SBSS degradation rate of C/GFRP rods was fast during the initial stage of exposure, because water molecules interacted with the resin matrix, resulting in plasticization of the resin matrix. During the middle and late stages of exposure, the degradation rate of SBSS became slow, because the water absorption of C/GFRP rods reached saturation. For the same exposure time, the decrease in shear strength was significantly accelerated by temperature. Therefore, exposure temperature was the main factor affecting the interfacial strength of C/GFRP rods.

### 3.3. Degradation of the Dynamic Thermal Mechanical Properties

**Figure 11:**
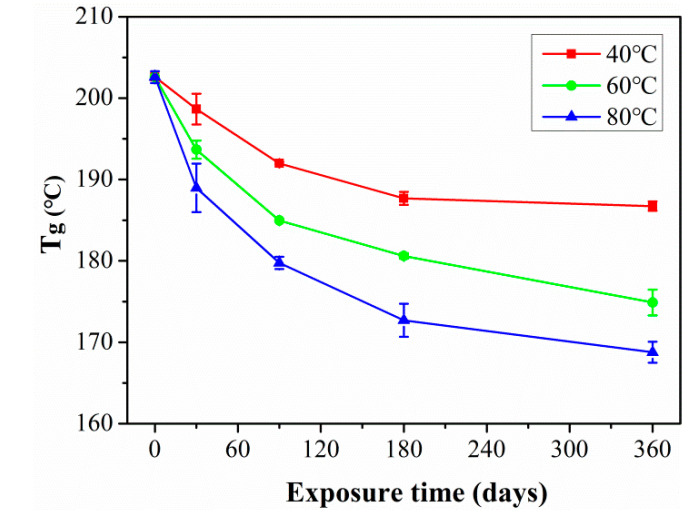
The changes of T_g_ exposed at 40 °C, 60 °C and 80 °C with exposure time.

Figure 11 shows the changes in glass transition temperature (T_g_) of the C/GFRP rod with exposure time. As the exposure time increased to 360 days, the water molecules gradually diffused from the C/GFRP cortex to the core layer and further entered the internal pores of the fiber–resin interface and resin matrix, resulting in resin hydrolysis, plasticization, and fiber–resin interface debonding, which led to a decrease in T_g_. Furthermore, at this stage, the higher exposure temperature accelerated the destruction of the water molecules, resulting in a significant decrease in T_g_ with the increase in exposure temperature. However, it was noted that the T_g_ of C/GFRP rods tended to increase in the later stage (360 days) of exposure at 40 °C. This was because the positive post-curing effect of the resin matrix was greater than the negative degradation influence of the fiber–resin interface, resin hydrolysis, and plasticization. For the 60 °C and 80 °C exposure environments, the exact opposite occurred. This was because the invasion of water molecules replaced the hydrogen bond between the molecular chains, increased the freedom of molecular chain movement, and significantly improved the movement of molecular chains. Similar to the degradation of SBSS, the degradation rate of T_g_ slowed down as the water absorption approached saturation.

**Figure 12:**
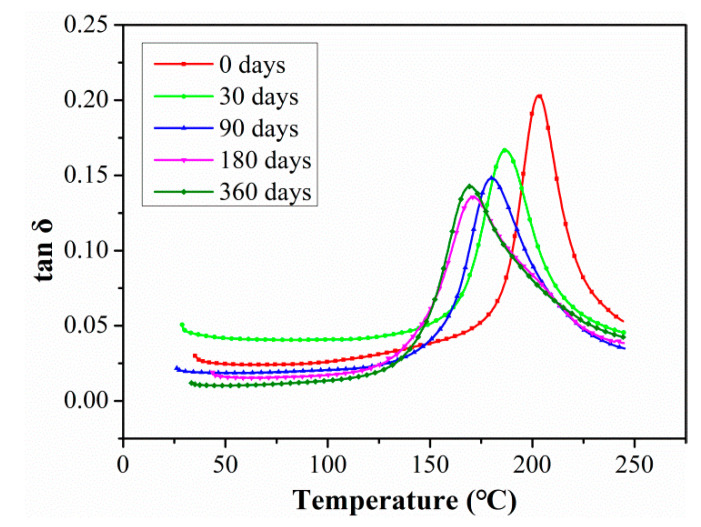
The changes of tanδ curves of C/GFRP rod exposed at 80 °C with aging time.

Figure 12 shows the changes in tanδ curves of C/GFRP rods with aging time under the exposure condition of 80 °C. Previous studies [34,35] have shown that there was a linear relationship between the horizontal crosslinking of the resin matrix and loss tangent profile; that is, the increase in loss tangent profile indicated the improvement in the horizontal crosslinking of the resin matrix. As shown in Figure 12, the curve peak of tanδ decreased with exposure time from 30 days to 360 days compared with the unaged sample, especially during the initial stage. This was because the ingression of water molecules in the initial stage led to significant resin plasticization, which reduced the cross-linking density between the molecular chains and then decreased the damping property of the resin matrix. Additionally, the ingression of a large number of water molecules into C/GFRP rods led to obvious interfacial debonding, which reduced the restriction of the interface on the molecular chain of the resin matrix, and greatly improved the freedom of movement of the molecular chain and the damping performance of the resin matrix. In addition, the horizontal axis of the tanδ peak shifted to the left after prolonged exposure, indicating T_g_ degradation in the C/GFRP rods.

**Figure 13:**
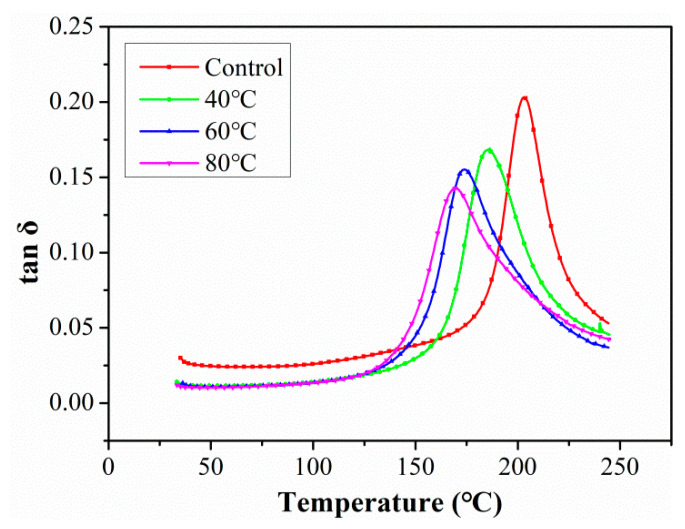
The changes in tanδ curves of the C/GFRP rod after 360 days of exposure with different aging temperatures.

After 360 days of exposure, the changes in tanδ curves according to exposure temperature are shown in Figure 13. It was found that exposure temperature had a significant effect on the peak of the tanδ curve, especially in the 80 °C immersion environment. This was because the C/GFRP rods reached saturation after exposure for 360 days at all of the immersion temperatures, and the fiber–resin interface underwent severe degradation under high temperature immersion conditions. Furthermore, the horizontal coordinate of the peak tanδ gradually shifted to the left with exposure temperature, which again suggested that the elevated temperature increased with the decrease in T_g_.

### 3.4. Scanning Electron Microscopy

**Figure 14:**
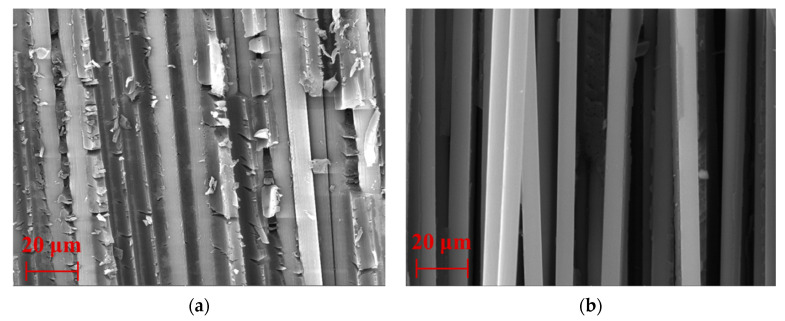
The fracture topography of short-beam shear for the control and aged specimens: (**a**) control and (**b**) 80 °C.

Figure 14 shows the shear fracture morphologies of the short-beam shear on C/GFRP rods. As seen in Figure 14a, the fibers were covered with a large amount of residual resin for the unaged C/GFRP rod, while the fibers were more uniformly embedded within the resin, indicating a strong bond at the fiber–resin interface. As seen in Figure 14b, the resin matrix was heavily hydrolyzed and the fibers were completely exposed for the aged C/GFRP rod, which indicated that the transverse restraint between the fibers was lost. This was because the ingression of water molecules in the elevated temperature exposure seriously etched the resin matrix inside the C/GFRP rod, resulting in serious hydrolysis of the resin polymer chain, which conformed to the law of obvious decrease in SBSS after exposure. In summary, long-term hygrothermal exposure led to irreversible effects from fiber–resin interface debonding, which resulted in significant SBSS degradation in the C/GFRP rods.

### 3.5. The Long-Term Life Prediction of Short-Beam Shear Properties

#### 3.5.1. Arrhenius Theory

The Arrhenius equation is an empirical formula for the relationship between the rate constant of chemical reactions and temperature created by Arrhenius from Sweden, as shown in Equation (6). The Arrhenius equation is based on the theory of statistical thermodynamics. In a chemical reaction, collisions between molecules of reactants need to reach a certain energy, namely the activation energy (*E_a_*), in order for the reaction to proceed. As the temperature increases, the average energy of the molecules increases and the proportion of molecules that can reach the activation energy increases, resulting in a faster reaction rate.

(6) $$k=A\exp(-E_{a}/RT)$$

where *k* is the performances degradation rate, *A* is constant, *E_a_* is activation energy, *R* is universal gas constant, and *T* is absolute temperature.

According to Equation (6), Arrhenius assumed that the increase in temperature would speed up the chemical reaction rate, and not change the chemical reaction mechanism. The time required for the same chemical reaction at different temperatures satisfied the following relationship.

(7) $$TSF=\frac{t_{0}}{t_{1}}=\frac{k_{1}}{k_{0}}=\frac{A\;\exp(-E_{a}/RT_{1})}{A\exp(-E_{a}/RT_{0})}=\exp\left[\frac{E_{a}}{R}(\frac{1}{T_{0}}-\frac{1}{T_{1}})\right]$$

where *t*_0_ and *t*_1_ are the required time under the temperature of *T*_0_ and *T*_1,_ respectively, and the ratio of *t*_0_/*t*_1_ is the time-shift factor (*TSF*).

The Arrhenius equation has a wide range of applications in several fields, such as chemistry, biology, and materials science. It can be used not only to study the relationship between a single reaction rate and temperature, but also to evaluate the effect of catalysts, reaction conditions, and other factors on the reaction rate. In the field of materials science, the Arrhenius equation helps to reveal the effect of temperature on the composite properties and provides theoretical support for composite property prediction and optimization.

#### 3.5.2. Life Prediction of SBSS

Based on the Arrhenius equation, many scholars have proposed prediction methods for the shear strength degradation of FRP composites [36]. Phani et al. [37] showed that the interfacial strength of FRP composites decreased to stable level and converged to a constant with the increase in exposure time. The detailed degradation model of shear strength is shown in Equation (8).

(8) $$Y_{t}=\left(100-Y_{\infty }\right)\exp(-t/\tau )+Y_{\infty }$$

where *Y_t_* is the retention of SBSS, *t* is exposed time, *τ* is the fitted parameter, and *Y*_∞_ is the final strength retention of SBSS.

**Figure 15:**
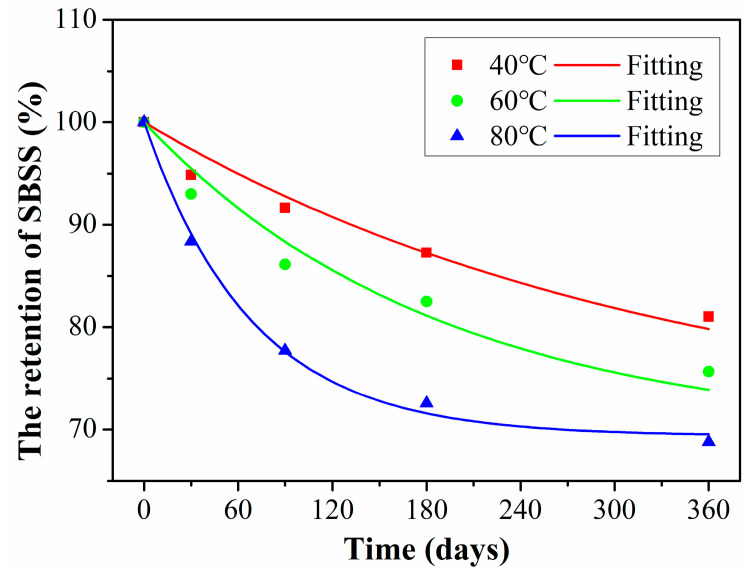
Long-term prediction curves of SBSS exposed at 40 °C, 60 °C and 80 °C.

Furthermore, the nonlinear fit with Equation (8) can be used to obtain the long-term prediction results of hybrid rods according to the related research [38], the fitted curves of SBSS at three exposure temperatures were obtained, as shown in Figure 15 and the fitted parameters are shown in Table 5. It was found to be well fitted between the fitting curve and the experimental data, which indicates that the degradation of SBSS satisfied the Arrhenius acceleration theory. In addition, the fitted parameter of *τ* decreased with exposure temperature, which indicates the exposure temperature had a significant effect on the SBSS degradation rate.

In order to obtain the *TSF* between the laboratory aging temperature and the actual service temperature, the activation energy *E_a_* for SBSS degradation should be obtained first. Furthermore, by transforming Equation (6) into Equation (9), and then placing the fitted parameters *τ* and *Y*_∞_ into Equation (9), time *t* can be obtained when SBSS reached 84%, 88%, 92%, and 96% retention at 40 °C, 60 °C, and 80 °C, respectively.

(9) $$\ln\left(\frac{1}{k}\right)=\frac{E_{a}}{R}\frac{1}{T}-\ln A$$

**Figure 16:**
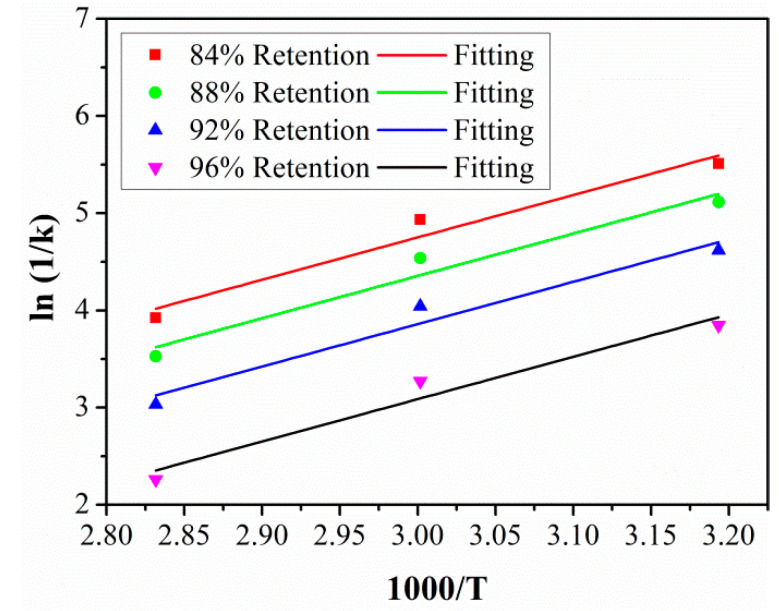
Arrhenius plots of SBSS retention.

According to Equation (9), the scatter points of ln (1/*k*) and 1000/*T* were plotted, and linear fitting was used to establish the linear relationship between ln (1/*k*) and 1000/*T*. As shown in Figure 16, the fitting curves fitted well with the scatter points, and the fitting curves were parallel with each other; this also proved that the degradation of SBSS under a hygrothermal environment conformed to the Arrhenius theory. The fitted parameters of *E_a_*/*R* are listed in Table 6. It should be noted that the slope of *E_a_*/*R* represents the energy barrier of SBSS degradation and the lower value of *E_a_*/*R* represents the high degradation rate of SBSS.

Then, by substituting *E_a_*/*R* in Table 6 into Equation (7), the *TSF* between the laboratory aging temperature and the actual service temperature could be calculated. In the present paper, three actual service temperatures of 8.1 °C, 15.8 °C, and 21.8 °C were selected, referencing the research of [39], in order to conduct the long-term life prediction of SBSS. The calculated *TSF* are listed in Table 7.

**Figure 17:**
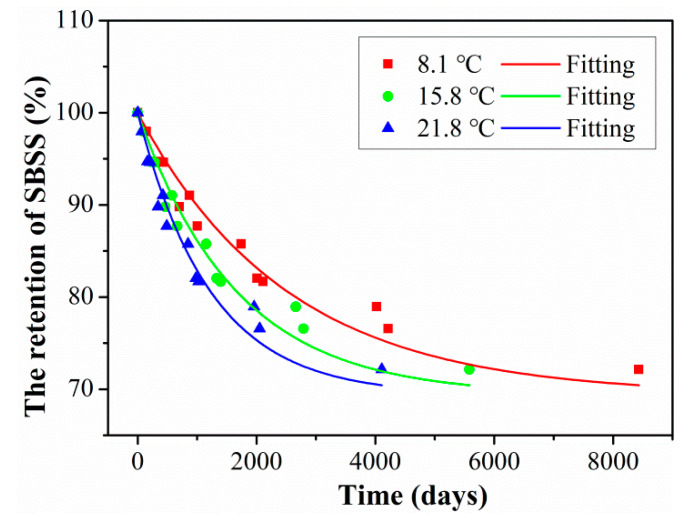
Long-term life decrease curves of SBSS for three actual service temperatures.

Arrhenius assumed that the increase in temperature would speed up the chemical reaction rate and not change the chemical reaction mechanism. The exposure time (abscissa in Figure 10) in the laboratory multiplied by the time-transfer factor (TSF) obtained the required time at the actual service temperature for the hybrid rod. Furthermore, the long-term life prediction curves could be obtained through the above prediction model of Equation (8), and the prediction results are shown in Figure 17 and the detailed fitted parameters are listed in Table 8. As seen in Figure 17, the SBSS decreased rapidly at the initial aging stage, and then the degradation rate tended to slow down. In addition, a higher exposure temperature increased the degradation rate of SBSS in the initial stage and decreased the time taken for SBSS to reach stable retention. The long-term life retention of SBSS reached a stable level of 69.38%, which is a meaningful design guideline for civil engineering structures.

In order to quantitatively analyze the degradation rate during the initial exposure of the hybrid rods, the required service time for SBSS to reach 90% retention was obtained, and the detailed results are listed in Table 9. Furthermore, the service times when the retentions of SBSS decreased to 90% were 2657 days, 1758 days, and 1294 days for the exposure temperature of 8.1 °C, 15.8 °C, and 21.8 °C, respectively. It can be seen that the initial degradation rate of SBSS was increased by the service temperature.

## 4. Conclusions

The water absorption behaviors of C/GFRP rods in a water immersion environment were experimentally studied, the changes of their mechanical properties were analyzed, and establishing a life prediction model was attempted. Based on the experimental results, this paper explored the effect of water absorption on the mechanical properties, and then developed a life prediction model to obtain the life evaluation of C/GFRP rods. This study aimed to provide support for the life evaluation and design parameters for C/GFRP composites in practical applications. The detailed conclusions are as follows.

(1) The water absorption of the hybrid rod conformed to the classical fickle diffusion model. The water absorption concentration was determined by the radial position, exposure temperature, and time. In addition, the radial position of the water molecules diffused into the rod was positively correlated with the diffusion concentration.

(2) The short-beam shear strength of the hybrid rod decreased significantly after 360 days of exposure, because the water molecules combined with the resin matrix through hydrogen bonds to form bound water during the immersion process, which led to the hydrolysis and plasticization of the resin matrix and the debonding of the fiber–resin interface.

(3) After 360 days of exposure, the T_g_ of the C/GFRP rods decreased by 6.9% (40 °C), 13.3% (60 °C), and 17.4% (80 °C), respectively, because the ingression of water molecules led to degradation in the viscoelastic behavior of C/GFRP rods.

(4) The Arrhenius equation was used to obtain the long-term life of SBSS in actual service temperature based on the time–temperature equivalence theory, in which the stable strength retention of SBSS was 69.38%, which is a meaningful durability design parameter for hybrid rods in civil engineering structures.

## Figures and Tables

**Table 1:** The mechanical properties of the fibers and resin.

Mechanical Properties	Carbon Fiber	Glass Fiber	Resin
Tensile modulus (GPa)	230	85	2.50
Tensile strength (MPa)	4900	2720	112.5
Elongation (%)	2.13	3.2	4.50

**Table 2:** The fitting parameters of the water absorption parameters for the C/GFRP rods (*M*_∞_, *D*). *R*^2^ is the correlation coefficient used to characterize the fitting degree.

Temperature (°C)	Fitting Parameters		
	*M*_∞_ (%)	*D* (×10^−13^ m^2^/s)	*R^2^*
40	0.30	2.00	0.99
60	0.52	2.44	0.98
80	0.72	3.13	0.99

**Table 3:** Moisture distribution cloud images in C/GFRP rods at different times and temperatures.

Exposure Environment	40 °C	60 °C	80 °C
10 days	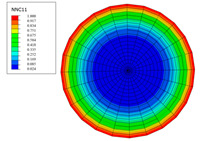	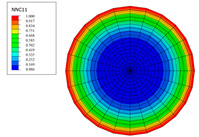	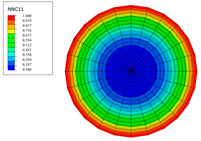
30 days	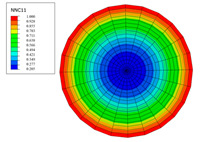	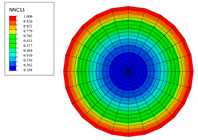	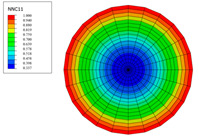
90 days	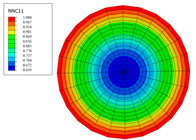	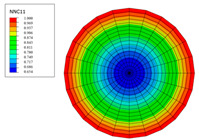	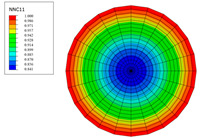

**Table 4:** The time (days) required for saturated water absorption at different radial positions and exposure temperatures.

Exposed Temperature (°C)	Radial Position (mm)			
	R = 0	R = 1	R = 2	R = 3
40	622.29	607.36	555.97	418.13
60	510.14	497.99	455.63	342.78
80	397.64	388.26	355.28	267.08

**Table 5:** Fitted parameters of SBSS for hybrid rod.

Temperature (°C)	Fitted Parameters		
	*τ*	*Y*_∞_ (%)	*R* ^2^
40	334.55	69.38	0.96
60	188.06	69.38	0.95
80	68.44	69.38	0.99

**Table 6:** Fitted parameters of Arrhenius plot for SBSS retention.

Retention (%)	*E_a_/R*	*R* ^2^
84	4357.79	0.98
88	4357.79	0.98
92	4357.79	0.98
96	4357.79	0.98

**Table 7:** *TSF* of SBSS exposed at actual service temperature of 8.140 °C, 15.8 °C and 21.8 °C.

Laboratory Temperature (°C)	Actual Service Temperature (°C)		
	8.1	15.8	21.8
40	6.57	4.01	2.78
60	17.79	10.87	7.54
80	43.05	26.31	18.24

**Table 8:** The fitted parameters of SBSS at actual service temperatures.

Service Temperature (°C)	*τ* (1/d)	*R* ^2^
8.1	2509.13	0.99
15.8	1660.42	0.99
21.8	1221.53	0.99

**Table 9:** Required time for SBSS reach to 90% retention.

Service Temperature (°C)	Service Time (Days)
8.1	2656.95
15.8	1758.24
21.8	1293.50

## Data Availability

Not applicable.
